# Energy Localization through Locally Resonant Materials

**DOI:** 10.3390/ma13133016

**Published:** 2020-07-06

**Authors:** Marco Moscatelli, Claudia Comi, Jean-Jacques Marigo

**Affiliations:** 1 Department of Civil and Environmental Engineering, Politecnico di Milano, Piazza Leonardo da Vinci 32, 20133 Milan, Italy; marco.moscatelli@polimi.it; 2 Laboratoire de Mécanique des Solides, École Polytechnique, Route de Saclay, 91120 Palaiseau, France; jean-jacques.marigo@polytechnique.edu

**Keywords:** locally resonant material, energy harvesting, homogenization

## Abstract

Among the attractive properties of metamaterials, the capability of focusing and localizing waves has recently attracted research interest to establish novel energy harvester configurations. In the same frame, in this work, we develop and optimize a system for concentrating mechanical energy carried by elastic anti-plane waves. The system, resembling a Fabry-Pérot interferometer, has two barriers composed of Locally Resonant Materials (LRMs) and separated by a homogeneous internal cavity. The attenuation properties of the LRMs allow for the localization of waves propagating at particular frequencies. With proper assumptions on the specific ternary LRMs, the separation of scales (between the considered wave lengths and the characteristic dimension of the employed unit cells) enables the use of a two-scale asymptotic technique for computing the effective behavior of the employed LRMs. This leads to a complete analytic description of the motion of the system. Here we report the results achieved by optimizing the geometry of the system for obtaining a maximum focusing of the incoming mechanical energy. The analytic results are then validated through numerical simulations.

## 1. Introduction

The conversion of vibrations into low-power electricity has been extensively explored since decades in view of the wide range of applications of the harvesters in distributed wireless sensors for structural health monitoring and for security systems, in embedded and implanted medical sensors and in recharging of batteries in various systems, see, e.g., the reviews [1,2]. To obtain an efficient energy harvester, one has to focus the mechanical energy produced by external vibrations in a given region of the system, where it can be then converted in electricity by means, e.g., of piezoelectric materials.

The use of structured materials for Energy Harvesters (EH) has more recently attracted the attention of the researchers and a number of possible configurations have been proposed [3,4,5,6,7]. Localized defects in phononic crystals [8] and in micro-structured plates [9] have been exploited to focus vibration energy in a small region where a piezoelectric material converts the mechanical energy into the electric one. However, good performance is obtained only for frequencies close to the defect eigen-frequency which is related with the typical size of the unit cell of the lattice. Even though proper methods can be used to optimize the geometry of the cell [10], for small devices, this frequency is very high when compared with the frequency of ambient vibrations in common applications. Localized defects in tensegrity materials have also been studied in [11].

Locally resonant metastructures, which are structures that comprise Locally Resonant Material (LRM) components, enable band gap formation at wavelengths much longer than the lattice size (see, e.g., [12,13,14,15,16]), therefore they can represent good candidates for efficient, small EH. In [17] the localized deformation pattern achieved in the frequency neighborhood of the band gaps was employed in order to harvest a certain amount of the kinetic energy available in the oscillating members of the lattice. Recently [18] proposed a prototype of locally resonant energy harvesting-metastructure composed of a primary beam with several small secondary cantilever beams with tip masses acting as mechanical resonators. The design of innovative metasurfaces, with graded resonators, that trap waves was proposed and developed in [5] for enhanced piezoelectric energy harvesting.

In the present work we explore the possibility to couple the advantage of the LRM mechanism to create band gaps at low frequencies with the energy localization mechanism in local defects of regular lattices to design and optimize a Resonant Energy Harvester (REH). An initial attempt in this direction was presented in [19], considering binary LRMs. The ternary LRM here considered, endowed with two-dimensional periodicity, is constituted by a stiff matrix with almost rigid cylindrical inclusions coated by a compliant material. The two-scale homogenization approach, proposed in [20] for high-contrast binary composite materials in the long wavelength regime and developed by many authors ([21,22,23]), provides a powerful tool to define equivalent material properties. The authors recently studied through homogenization the spectral properties and the band gaps of binary and ternary LRM in [16,24], respectively. The REH here proposed is constituted by two barriers made of the above LRM and a cavity, made of the matrix alone, which represents a line defect in the two dimensional regular lattice. The use of barriers made of LMR and of phononic crystals was also recently proposed to obtain an acoustic diode [25]. To optimize the REH, the behavior of the LRM is here characterized by its homogenized properties and the dynamic response is analytically obtained. The conditions to obtain the maximal mechanical energy inside the cavity are explicitly given and the role of the different configurations of the unit cell of the metamaterial are highlighted.

Beside extending the analytic results obtained in [19] for a REH with binary LRM to the case of a REH with three components LRM, the paper provides a new numerical validation, introduces the concept of indexes of concentration and develops maps of these indexes at varying frequency and geometry of the REH. The maps can be of practical use in the design of a real REH that, with a fixed geometry, should work in a wide range of frequencies.

The paper is organized as follows. The proposed configuration of the REH and the dynamic problem of shear wave propagation is set in Section 2. In Section 3 the energy in each part of the system is computed and the optimal width of the cavity to maximize the energy trapped in the cavity is obtained. Section 4 provides a comparison of the analytic results with the numerical results obtained for a special choice of the LRM, thus validating the analytic results. These latter allows to perform a parametric study of the system. Some conclusions are given in Section 5. The results of the asymptotic homogenization, obtained in [24], which are used in the paper, are recalled in the Appendix A.

Notation, vectors and tensors are indicated by bold face letters. Complex numbers are denoted with sans serif letters, while italic is utilized for the Real numbers. The complex conjugate of a complex number is indicated by a superposed bar. The angular frequency ω is often called just frequency. The notation 〈·〉 denotes the average over the period.

## 2. The Resonant Cavity in Metamaterials

### 2.1. Problem Formulation

**Figure 1:**
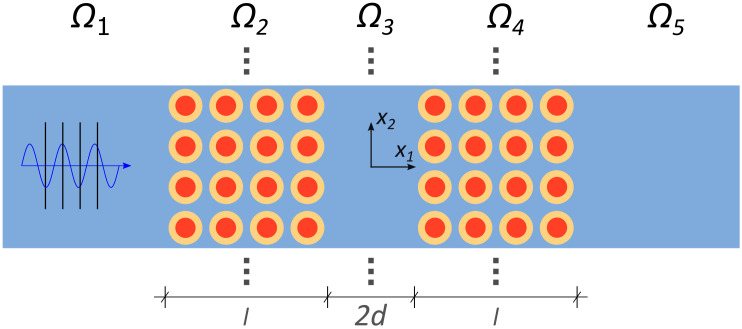
Top-view of the studied system. The wave shown in the figure represents an out-of-plane wave propagating throughout all the five domains. The dots denote the fact that the system must be extended along the $x_{2}$ direction.

We consider the system represented in Figure 1. It is composed of five domains: parts $\Omega_{1}$, $\Omega_{3}$ and $\Omega_{5}$ are constituted by an isotropic homogeneous medium (defined as $\Omega_{h}$ in the following), whereas parts $\Omega_{2}$ and $\Omega_{4}$, which will be denoted as “barriers” ($\Omega_{b}$), are made of the same LRM. This system, which is similar to a Fabry-Pérot interferometer in optics [26], will be referred to as Resonant Energy Harvester (REH). A similar configuration has been already studied by the authors in [19], although now the two barriers are made of a ternary LRM instead of the binary heterogeneous material previously employed.

**Figure 2:**
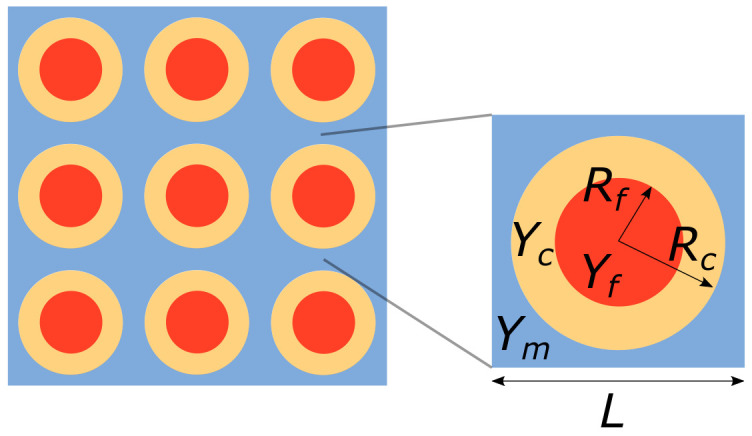
Lattice of the LRM and zoom over a unit cell composing it. $R_{f}$ and $R_{c}$ are respectively the fiber and coating external radii.

The LRM is composed of a two dimensional periodic repetition of the square unit cell represented in Figure 2, with a stiff matrix $Y_{m}$ containing an almost rigid circular fiber $Y_{f}$, coated by a very compliant layer $Y_{c}$. The developed system has a thickness in the out-of-plane direction and along the axis $x_{2}$ which is much larger than the characteristic size *L* of the unit cell; parts $\Omega_{1}$ and $\Omega_{5}$ are infinitely extended towards −∞ and +∞ along the axis $x_{1}$.

We analyze the propagation of anti-plane waves along the $x_{1}$ direction, allowing for the decoupling from the in-plane wave propagation problem. In the harmonic regime, this results in the following two dimensional Helmholtz scalar equation:

(1) $$\left\{\begin{array}{c} \mathrm{div}\sigma +\rho \;\omega^{2}u=0,\;\sigma =\mu \;\;\mathrm{grad}\;u\\ \mathrm{with}\;\;u(x,t)\;\;\mathrm{and}\;\;\sigma \cdot n\;\;\mathrm{continuous}\;\mathrm{at}\;\mathrm{each}\;\mathrm{interface} \end{array}\right.$$

where the elastic displacement u(x,t) has a time dependence $\;e^{i\omega t}$, which from now on will be omitted and σ collects the non vanishing stress components $\sigma_{31}$ and $\sigma_{32}$. The mass density ρ and the shear elastic modulus μ are spatially varying parameters, depending on the position x in the system, as specified in the following:

(2) $$\mu =\left\{\begin{array}{cc} \mu_{m} & \;\mathrm{in}\;Y_{m}\\ \mu_{f} & \;\mathrm{in}\;Y_{f}\\ \mu_{c} & \;\mathrm{in}\;Y_{c} \end{array}\right.,\;\rho =\left\{\begin{array}{cc} \rho_{m} & \;\mathrm{in}\;Y_{m}\\ \rho_{f} & \;\mathrm{in}\;Y_{f}\\ \rho_{c} & \;\mathrm{in}\;Y_{c} \end{array}\right.$$

Denoting by $k_{m}=\omega \sqrt{\rho_{m}/\mu_{m}}$ the wave number in the matrix, we limit our study by considering a low frequency regime such that the dimensionless parameter $\epsilon =k_{m}L$ be very small (ϵ≪1). This assumption can be equivalently set by requiring that the characteristic size *L* of the cell be much smaller than the wave length, in the matrix, of the considered wave. This hypothesis enables the application of a two-scale asymptotic homogenization technique for the description of the motion of the barriers. By assuming $\rho_{m}/\rho_{c}=O\left(1\right)$ and the ratio $\mu_{c}/\mu_{m}$ of order $O(\epsilon^{2})$ the effective mass density $\rho_{eff}$ and the effective shear modulus $\mu_{eff}$ of the ternary LRM were computed in [24] through homogenization. In the Appendix A we summarize the main results derived in [24], which are used for treating the problem here analyzed. The reader is thus addressed to that paper for a more complete derivation.

### 2.2. Solution of the Homogenized Problem

**Figure 3:**
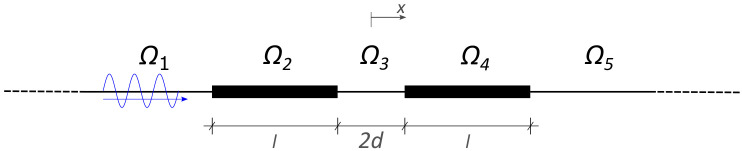
One-dimensional counterpart of the two-dimensional homogenized problem. The system is simplified by fixing a position along the $x_{2}$ axis and by considering $x_{1}=x$. The dashed ends are used to indicate that the domain is infinitely extended toward −∞ and +∞.

By virtue of the homogenization, the REH can be treated as an equivalent system composed of five homogeneous and isotropic parts. Since we are interested in the propagation of waves whose wave front are perpendicular to the $x_{1}$ direction, there is no dependence on the $x_{2}$ direction, $\sigma_{32}=0$ and the problem becomes one-dimensional, obtaining the simplified system sketched in Figure 3 (from now on thick lines will represent a LRM).

To summarize, we report here below the homogenized problem governing the motion inside each of the five parts composing our system:

(3) $$\left\{\begin{array}{cc} \mathrm{div}\sigma \left(x\right)+\rho_{m}\;\omega^{2}U\left(x\right)=0,\;\sigma =\mu_{m}\;\;\partial U\left(x\right)/\partial x & \;\mathrm{in}\;\Omega_{h}\\ \mathrm{div}\sigma \left(x\right)+\rho_{eff}\left(\omega \right)\;\omega^{2}\;U\left(x\right)=0,\;\sigma =\mu_{eff}\;\;\partial U\left(x\right)/\partial x & \;\mathrm{in}\;\Omega_{b}\\ \mathrm{with}\;\;U(x)\;\;\mathrm{and}\;\;\sigma (x)\;\;\mathrm{continuous}\;\mathrm{at}\;\mathrm{each}\;\mathrm{interface} \end{array}\right.$$

where U(x) is the first term in the expansion of the displacement field in the matrix (denoted by $U^{0}\left(x\right)$ in the Appendix A) and $\sigma =\sigma_{31}$ denotes the only non-zero stress component.

As specified in the Appendix A, the sign of the effective mass density $\rho_{eff}$ depends whether the frequency ω is inside or outside a band gap; this means that within the barriers $\Omega_{2}$ and $\Omega_{4}$ the second of the Equation (3) has a general solution whose form varies depending on the frequency, as follows:

(4) $$\left\{\begin{array}{cc} U_{j}\left(x\right)=A_{j}\;\;e^{-isx}\;+\;B_{j}\;\;e^{isx} & \;\mathrm{when}\;\rho_{eff}\geq 0\\ U_{j}\left(x\right)=A_{j}\;\cosh sx\;+\;B_{j}\;\sinh sx & \;\mathrm{when}\;\rho_{eff}<0 \end{array}\right.$$

where $s=\omega \;\sqrt{\frac{|\rho_{eff}|}{\mu_{eff}}}$, $A_{j}$ and $B_{j}$ (with j=2,4) are complex integration constants.

The general solution of the motion problem inside the regions $\Omega_{h}$ is instead always given by:

(5) $$U_{j}\left(x\right)=A_{j}\;\;e^{-ikx}\;+\;B_{j}\;\;e^{ikx}$$

with j=1,3 or 5 and $k=k_{m}=\omega \;\sqrt{\frac{\rho_{m}}{\mu_{m}}}$.

Considering a propagating input wave, the integration constants are obtained imposing the continuity of the displacement and of the stress between the various regions and the conditions at infinite. In particular, when considering an incoming wave which travels from the left toward the right (see Figure 3) of amplitude 1, the displacement in the different parts $\Omega_{i}$ reads (see [19] for details):

(6) $$\left\{\begin{array}{c} U_{1}\left(x\right)=\;e^{-ik(x+d+l)}\;+\;R\;\;e^{ik(x+d+l)}\\ U_{2}\left(x\right)=\left(1+R\right)\cosh s(x+d+l)\;+\;i\;a\;\left(R-1\right)\;\sinh s(x+d+l)\\ U_{3}\left(x\right)=\frac{T}{2}\;\beta \;\;e^{ik(x-d)}+\frac{T}{2}\;\alpha \;\;e^{-ik(x-d)}\\ U_{3}\left(x\right)=T\;\cosh s(x-d-l)\;-\;i\;a\;T\;\sinh s(x-d-l)\\ U_{5}\left(x\right)=T\;\;e^{-ik(x-d-l)} \end{array}\right.$$

where R and T are respectively the amplitudes of the reflected and transmitted wave:

(7) $$\begin{array}{cc} T & =\frac{4}{\alpha^{2}\;\;e^{2ikd}-\beta^{2}\;\;e^{-2ikd}} \end{array}$$

(8) $$\begin{array}{cc} R & =\frac{\alpha \beta \;e^{2ikd}-\bar{\alpha }\bar{\beta }\;e^{-2ikd}}{\alpha^{2}\;\;e^{2ikd}-\beta^{2}\;\;e^{-2ikd}} \end{array}$$

with

(9) $$a=\frac{\mu_{m}k}{\mu_{eff}s},\;\alpha =2\cosh sl\;+\;i\left(a-\frac{1}{a}\right)\sinh sl,\;\beta =i\left(a+\frac{1}{a}\right)\sinh sl$$

## 3. Energy Localization in the Cavity

### 3.1. Energy in the Homogeneous Parts

We are now interested in finding the mechanical energy density, averaged over a period, along the whole REH 〈e(x,t)〉 and the total energy in parts $\Omega_{2}$, $\Omega_{3}$ and $\Omega_{4}$ again averaged over a time period, 〈E(t)〉.

The mechanical energy in regions $\Omega_{j}$ with j=1, 3 or 5 of homogeneous material, given by the sum of the potential energy *p* and the kinetic energy *c*, reads:

(10) $$e_{j}\left(x,t\right)=p_{j}+c_{j}=\frac{1}{2}\mu_{m}u_{j}^{\prime^{2}}+\frac{1}{2}\rho_{m}{\dot{u}}_{j}^{2}$$

where the dependency over time is again accounted for, a superimposed dot denotes the time derivative and a prime denotes the space derivative; the displacement field $u_{j}$ is given by $u_{j}\left(x,t\right)=Re\left\{U_{j}\left(x\right)\;\;e^{i\omega t}\right\}$.

Averaging Equation (10) over time gives:

(11) $$\langle e_{j}\rangle =\frac{\mu_{m}\;k^{2}}{2}\;\left[|A_{j}{|}^{2}+{\left|B_{j}\right|}^{2}\right]$$

Notice that $\langle e_{j}\rangle$ is independent from the position x. Finally, the total mechanical energy 〈E(t)〉 inside part $\Omega_{3}$ reads:

(12) $$\langle E_{3}\rangle =2d\;\langle e_{3}\rangle$$

While the energy inside the regions $\Omega_{h}$ can be easily obtained, a particular treatment must be devoted to the calculation of the energy inside the two barriers.

### 3.2. Energy Inside the LRM: Homogenization Approach

Each cell is composed of a matrix, a coating layer and a fiber considered as rigid, hence the mechanical energy density of one cell reads:

(13) $$e_{j}\left(x,t\right)=p_{j}^{m}+c_{j}^{m}+p_{j}^{c}+c_{j}^{c}+c_{j}^{f}$$

The homogenization technique enables to find the displacement field and the stress over a cell composing the metamaterial. It should be noted that, while at the macro-scale the problem is one-dimensional, so that only the variable $x_{1}=x$ must be retained, at the micro-scale (inside each cell) the problem remains 2D and the coordinates $y=y_{1},y_{2}$ or the polar coordinates r,ϑ must be retained. Similarly, inside the cell the stress $\sigma_{32}$ is non-zero and a stress vector should be considered. At the leading order, one has:

(14) $$u\left(x,t\right)≃u^{0}=\left\{\begin{array}{cc} U\left(x\right)\;e^{i\omega t}\; & \;\mathrm{in}\;Y_{m}\\ U\left(x\right)\eta \left(r\right)\;e^{i\omega t}\; & \;\mathrm{in}\;Y_{c}\\ U\left(x\right)\eta \left(R_{f}\right)\;e^{i\omega t}\; & \;\mathrm{in}\;Y_{f} \end{array}\right.$$

and

(15) $$\sigma \left(x,t\right)≃\sigma^{0}=\left\{\begin{array}{cc} \mu_{m}\left({\mathrm{grad}}_{y}\chi +e_{1}\right)\frac{\partial U(x)}{\partial x}\;e^{i\omega t}\; & \;\mathrm{in}\;Y_{m}\\ 0\; & \;\mathrm{in}\;Y_{c} \end{array}\right.$$

with η(r) and χ defined in the Appendix A.

Considering a frequency inside the band gap of the metamaterial, in regions $\Omega_{j}$, j=2,4, one has $U\left(x\right)=U_{j}\left(x\right)$, j=2,4, with $U_{j}$ given by Equation (6). Denoting the real part of the displacement in the matrix by $u_{j}\left(x,t\right)=Re\left\{U_{j}\left(x\right)\;\;e^{i\omega t}\right\}$ the contributions to the energy density in Equation (13) read

(16) $$\begin{array}{cc} p_{j}^{m} & =\frac{1}{2}\;\mu_{eff}\;u_{j}^{\prime^{2}} \end{array}$$

(17) $$\begin{array}{cc} c_{j}^{m} & =\frac{|Y_{m}|}{\left|Y\right|}\frac{\rho_{m}}{2}\;{\dot{u}}_{j}^{2} \end{array}$$

(18) $$\begin{array}{cc} p_{j}^{c} & =\frac{\mu_{c}}{2}\int_{Y_{c}}{\mathrm{grad}}_{y}\eta \cdot {\mathrm{grad}}_{y}\eta \;dy\;\frac{u_{j}^{2}}{\left|Y\right|} \end{array}$$

(19) $$\begin{array}{cc} c_{j}^{c} & =\frac{\rho_{c}}{2}\int_{Y_{c}}\eta^{2}\;dy\;\frac{{\dot{u}}_{j}^{2}}{\left|Y\right|} \end{array}$$

(20) $$\begin{array}{cc} c_{j}^{f} & =\frac{\rho_{f}}{2}\eta {\left(R_{f}\right)}^{2}\;\frac{|Y_{f}|}{\left|Y\right|}\;{\dot{u}}_{j}^{2} \end{array}$$

From Equations (16) to (20) after some manipulation, the energy $\langle e_{j}\rangle$, with j=2 or 4, can be written as:

(21) $$\begin{array}{c} \begin{array}{cc} \langle e_{2}\rangle =\frac{1}{4}\{\mu_{eff}\;s^{2} & {\left|\left(1+R\right)\sinh sl+a\;i\left(R-1\right)\cosh sl\right|}^{2}\;+\\ \; & \omega^{2}\gamma \;{\left|\left(1+R\right)\cosh sl+a\;i\left(R-1\right)\sinh sl\right|}^{2}\} \end{array} \end{array}$$

(22) $$\begin{array}{c} \begin{array}{cc} \langle e_{4}\rangle =\frac{1}{4}\{\mu_{eff}\;s^{2} & {\left|T\sinh sl-a\;iT\cosh sl\right|}^{2}\;+\\ \; & \omega^{2}\gamma \;{\left|T\cosh sl-a\;iT\sinh sl\right|}^{2}\} \end{array} \end{array}$$

where γ is given by:

(23) $$\gamma =\rho_{eff}\;+\;\frac{2}{\left|Y\right|}\;\left[\rho_{c}\;\int_{Y_{c}}\eta^{2}-\eta \;dy\;+\;\rho_{f}\left|Y_{f}\right|\eta \left(R_{f}\right)\left(\eta (R_{f})-1\right)\right]$$

The averaged mechanical energy density for the barriers is thus dependent on the position *x* along them. Integrating over their thickness *l*, one can derive the averaged total mechanical energies $\langle E_{2}\rangle$ and $\langle E_{4}\rangle$ as:

(24) $$\begin{array}{cc} \langle E_{2}\rangle & =\frac{1}{4}\{\left[\frac{l}{2}\left(a^{2}-1\right)\left(\mu_{eff}s^{2}-\omega^{2}\gamma \right)+\frac{\sinh2sl}{4s}\left(a^{2}+1\right)\left(\mu_{eff}s^{2}+\omega^{2}\gamma \right)\right]+\\ \; & -2\;Re\left\{R\right\}\left[\frac{l}{2}\left(a^{2}+1\right)\left(\mu_{eff}s^{2}-\omega^{2}\gamma \right)+\frac{\sinh2sl}{4s}\left(a^{2}-1\right)\left(\mu_{eff}s^{2}+\omega^{2}\gamma \right)\right]+\\ \; & -a\;Im\left\{R\right\}\left(1-\cosh2sl\right)\left(\mu_{eff}s^{2}+\omega^{2}\gamma \right)\} \end{array}$$

(25) $$\begin{array}{cc} \langle E_{4}\rangle & =\frac{1}{4}{\left|T\right|}^{2}\left[\frac{l}{2}\left(a^{2}-1\right)\left(\mu_{eff}s^{2}-\omega^{2}\gamma \right)+\frac{\sinh2sl}{4s}\left(a^{2}+1\right)\left(\mu_{eff}s^{2}+\omega^{2}\gamma \right)\right] \end{array}$$

### 3.3. Optimal Cavity Width

Our objective is now to exploit the attenuation capabilities of the employed LRMs for obtaining a concentration of mechanical energy inside the cavity. Let us focus our attention on frequencies inside a band gap for the analyzed LRM.

Fixing the materials used for the system, the width *l* of the barriers and the frequency of the propagating wave, the modulus of the coefficient T given in Equation (7) is maximized and becomes equal to 1 for a discrete set of optimal cavity widths ${\tilde{d}}_{n},n\in N^{*}$ and, at the same time, |R| is null. The optimal cavity widths ${\tilde{d}}_{n}$ can be found from the following relation (see [19] for the details):

(26) $$\tan4kd\;=\;\frac{2\;\left[2\cosh sl\right]\;\left[\left(a-\frac{1}{a}\right)\sinh sl\right]}{{\left[\left(a-\frac{1}{a}\right)\sinh sl\right]}^{2}\;-\;{\left[2\cosh sl\right]}^{2}}$$

One should notice that Equation (26) gives also the set of *d* that minimize T.

Using the expression of the displacement $U_{3}$ into Equation (11), the total mechanical energy Equation (12) inside part $\Omega_{3}$ reads:

(27) $$\langle E_{3}\rangle =d\;\frac{\mu_{m}\;k^{2}}{4}\;\left|T\right|\left[{\left|\alpha \right|}^{2}+{\left|\beta \right|}^{2}\right]$$

From Equation (27), as both α and β are independent from *d*, the mechanical energy inside part $\Omega_{3}$ is maximized whenever T=1, i.e., when T is maximum.

When $d={\tilde{d}}_{n}$, the total energy of the regions $\Omega_{2}$, $\Omega_{3}$ and $\Omega_{4}$ reads:

(28) $$\left\{\begin{array}{cc} \langle E_{2}\rangle & =\langle E_{4}\rangle =\frac{1}{4}\left[\frac{l}{2}\left(a^{2}-1\right)\left(\mu_{eff}s^{2}-\omega^{2}\gamma \right)+\frac{\sinh2sl}{4s}\left(a^{2}+1\right)\left(\mu_{eff}s^{2}+\omega^{2}\gamma \right)\right]\\ \langle E_{3}\rangle & =d\;\frac{\mu_{m}\;k^{2}}{4}\;\left[{\left|\alpha \right|}^{2}+{\left|\beta \right|}^{2}\right] \end{array}\right.$$

### 3.4. Limit Case of the Well

**Figure 4:**
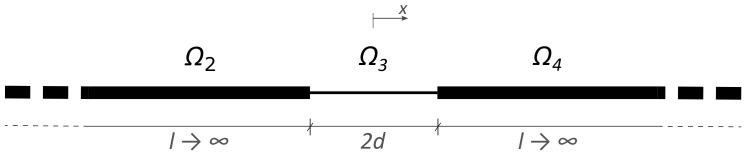
Sketch of the studied limit case. The barriers are infinitely extended toward −∞ and +∞.

By extending the barriers toward −∞ and +∞ the homogeneous parts $\Omega_{1}$ and $\Omega_{5}$ disappear and the above system tends to a well, as sketched in Figure 4.

The difference with respect to the REH case, expressed by Equation (3), is that now the two conditions at x→−∞ and x→+∞ are imposed to the metamaterial. Since no energy can be accumulated at +∞ and −∞, the motion inside parts $\Omega_{2}$ and $\Omega_{4}$ of the well must respect the following statements:

(29) $$U_{2}\left(x\to -\infty \right)=0\;U_{4}\left(x\to +\infty \right)=0$$

By applying the above conditions to the general solution of Equation (3), two of the six unknown integration constants can be given as functions of the remaining four. Furthermore, the continuity of displacements and stresses at each interface leads to a homogeneous system of four equations. This system admits a non-trivial solution only for a set of kd values, which can be found from the following relation:

(30) $$\tan2\;k\;d=\frac{2\;a}{a^{2}-1}$$

By fixing for instance the amplitude W of the left-wards traveling wave inside the well, the remaining three amplitudes are found and the motion inside each part is finally defined.

The only solutions are now modes for which the frequency is a resonant frequency given by Equation (30). Note that condition Equation (30) can also be obtained directly from Equation (26) by taking the limit for l→∞. The total mechanical energy stored by each part can be expressed as a function of |W| and reads:

(31) $$\begin{array}{cc} \langle E_{3}\rangle & ={2\;\theta \;|W|}^{2}\\ \langle E_{2}\rangle & =\langle E_{4}\rangle =\delta \;{\left|W\right|}^{2} \end{array}$$

with

(32) $$\theta =\mu_{m}k^{2}d,\;\delta =\frac{a^{2}}{2s(1+a^{2})}\left(\mu_{eff}s^{2}\;+\;\omega^{2}\gamma \right)$$

Fixing the amount of total energy of the system, one can find the coefficient |W| and, thus, the motion inside each part.

## 4. Results

The results of the previous sections are valid provided several hypotheses on the materials composing the REH are fulfilled. For clarity, we summarize them here below:

- Parts $\Omega_{1}$, $\Omega_{3}$ and $\Omega_{5}$ are constituted by the same material utilized for the matrix composing the LRM;
- The coating layer of the fibers must be very compliant with respect to the matrix;
- The fibers must be very stiff so that they can be treated as rigid in the homogenization procedure.

Since we are now interested in considering a real possible application, the material properties shall be fixed by respecting the three conditions just specified. We choose to use the following combination: an epoxy material is employed for the matrix, with lead inclusions coated by a layer of rubber. The material properties are specified in Table 1.

We consider a lattice with square unit cells with side L=1 mm, the radius of the coated circular inclusion is $R_{c}=0.415L$, while the radius of the internal part is $R_{f}=0.7R_{c}$. A scaling of the cell would simply scale the effective mass density when plotted with respect to the frequency, without changing the band gap structure; considering different filling fractions $\left(\pi R_{c}^{2}/L^{2}\right)$ or thicknesses of the coating, ($R_{c}-R_{f})$ would instead modify the dynamic behavior and the band structure (see [24] for other examples ).

### 4.1. Energy Localization: Analytic and Numerical Results

To check the validity of the analytic expressions derived in the previous sections, we have carried out some numerical analyses by using the commercial software COMSOL Multiphysics 5.4.

**Figure 5:**
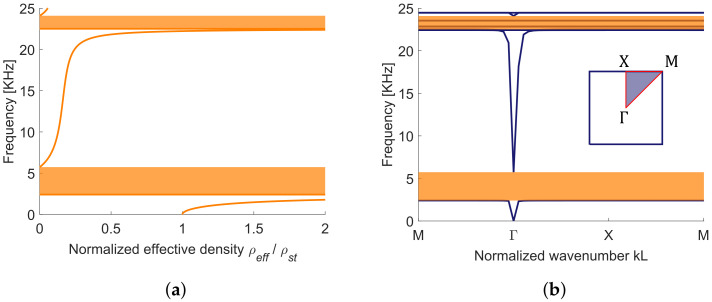
(**a**) Effective mass density vs. frequency, shaded areas correspond to negative effective mass, (**b**) dispersion plot with shaded band gaps obtained from the numerical Bloch-Floquet’s analysis; Irreducible Brillouin Zone and path followed for the numerical analysis is reported in the inset.

First of all, for the chosen material constants, from the homogenization technique we expect the presence of band gaps, as one can see from Figure 5a, where we have plotted the $\rho_{eff}$ for a range of frequencies ω from 0 to 25 kHz. The effective mass density becomes indeed negative between 2.4 and 5.7 kHz and between 22.5 and 24.1 kHz (within the range of frequencies shown by the plot). These intervals correspond to the band gaps obtained numerically by applying Bloch-Floquet’s periodic conditions at the unit cell boundaries of the LRM, as shown in Figure 5b. For the dispersion analysis, the cell is meshed with 2D triangular quadratic Lagrange finite elements and the formal analogy with the acoustic problem is exploited. The real stiffness of lead, see Table 1, is used, nonetheless the agreement with the analytic prediction, with the rigid inclusion assumption, is very good. One should remark that in the second band gap there are two flat modes which correspond to local resonances of the coated inclusion endowed with almost zero displacements in the matrix as already shown in [16].

Fixing to 40 the number of cells employed for each of the two barriers (hence fixing the thickness *l*) and optimizing the REH to work at a mid-band gap frequency $\tilde{\omega }=4.07$ kHz, Equation (26) gives a set of ${\tilde{d}}_{n}$, each of them giving a value of $\langle e_{3}\rangle$ 23 times bigger than the energy $\langle e_{in}\rangle$ carried by an incoming wave of unit amplitude. The energy of the incoming wave is obtained from Equation (11) and for a REH with the optimal cavity reads:

(33) $$\langle e_{in}\rangle =\frac{\mu_{m}\;k^{2}}{2}$$

The dynamic behaviour of this REH and the energy densities in the different domains were also numerically computed. In the numerical analyses, the actual two-dimensional system sketched in Figure 1 is considered. In the $x_{2}$ direction, only one row of the metamaterial is discretized and symmetry boundary conditions are imposed to simulate the ideal case. In the $x_{1}$ direction perfectly matched layers (PML) are added to consider the extension of the matrix towards infinity. 2D triangular quadratic elements are used to mesh the barriers, while 2D rectangular quadratic Lagrange finite elements are employed for the remaining homogeneous parts. Use is made again of the formal analogy with the acoustic problem, hence a background pressure field, analogous to the incoming out of plane wave, is imposed inside part $\Omega_{1}$ and the energy density is integrated in $x_{2}$ and in time over a period to be compared with the analytic results.

**Figure 6:**
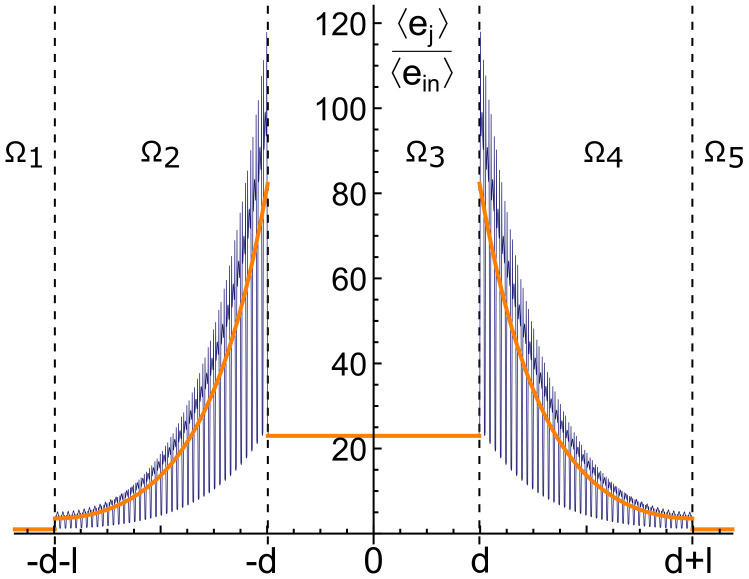
Ratio of the averaged mechanical energy density along the whole system, with respect to the incoming one. Orange: analytic results and blue: numerical results. Each part composing the REH is separated by the vertical dashed lines.

The plot in Figure 6 shows $\langle e_{j}\rangle /\langle e_{in}\rangle$ (with *j* from 1 to 5) along the whole system ${⋃}_{j=1}^{5}\Omega_{j}$ for ${\tilde{d}}_{1}=19.9$ mm. The orange lines represent the energy density computed by the analytic Equations (11) and (21), while the results of the numerical analysis are shown in blue. The oscillations of the numerical response are due to the intrinsic heterogeneity of the LRM composing the barriers, the analytic results, based on an homogenized material, give a mean value in good agreement.

**Figure 7:**
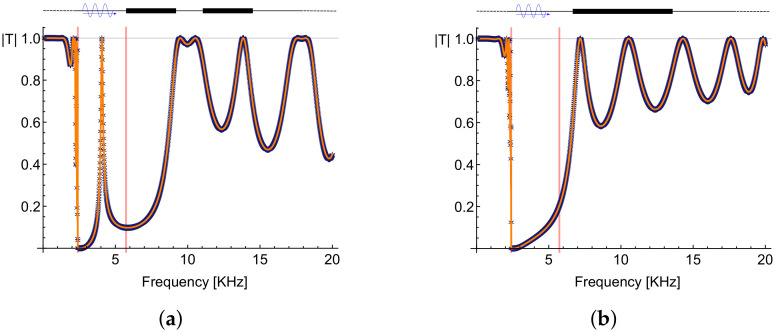
Transmission coefficient modulus |T| as a function of the frequency ω in presence of the cavity (**a**) and without any cavity (**b**). The vertical red lines define the band gap. Orange: analytic results and blue: numerical results of analyses in the frequency domain, considering a sweep of frequencies from 0 to 20 KHz.

In Figure 7a, we report the transmission coefficient modulus |T| vs. frequency, from a transmission analysis of our REH. The results coming from the asymptotic technique agrees very well with those of the real case numerically studied. The expected peak of transmission at the frequency $\tilde{\omega }$ inside the band gap is well captured both by the analytic and numerical results.

For showing how the presence of a cavity modifies this behavior, we have plotted in Figure 7b the results coming from a transmission analysis in absence of the cavity, i.e., for a simple layer of LRM with a thickness equal to 2l (obtained by attaching together the two barriers and getting rid of the cavity in the middle). Comparing these results with those in Figure 7a, it is clear how the peak inside the band gap disappears when no cavity is present.

### 4.2. Towards the Optimization of the Harvester: Parametric Study

The results of the previous subsection refer to a particular case, with all the parameters involved in the problem fixed. In this subsection we consider the effect of different possible configurations of the system and, to compare them, we introduce two indexes as a “measure” of the harvesting capabilities of the REH.

By keeping for the unit cell the same geometric dimensions employed in the previous subsection, there are three parameters which can be left free to vary, namely the barrier width *l*, the optimal cavity width ${\tilde{d}}_{n}$ and the frequency ω. To compare different configurations we introduce the following two indexes:

(34) $$\begin{array}{c} \begin{array}{cc} \mathrm{IC} & =\frac{\langle E_{3}\rangle }{\langle E_{2}\rangle +\langle E_{3}\rangle +\langle E_{4}\rangle }\\ \mathrm{AIC} & =\frac{\langle E_{3}\rangle /\left({\tilde{d}}_{n}\right)}{\left(\langle E_{2}\rangle +\langle E_{3}\rangle +\langle E_{4}\rangle \right)/\left({\tilde{d}}_{n}+l\right)} \end{array} \end{array}$$

where IC stands for “index of concentration” and AIC for “averaged index of concentration”. The former represents a measure of the concentration level of energy reached by the system, whereas the latter gives a ratio between values of energy averaged over the dimensions of the corresponding REH components.

**Figure 8:**
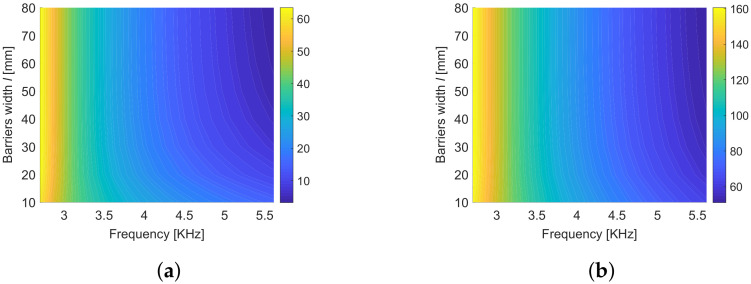
First (**a**) and second (**b**) optimal ${\tilde{d}}_{n}$ plotted with respect to the the frequency ω inside the first band gap and the width *l* of the barriers.

As stated previously, Equation (26) gives a set of optimal cavity widths $2{\tilde{d}}_{n},n\in N^{*}$, that maximize the focused energy for a given frequency inside the band gap. In the contours of Figure 8, we show the variation of the two smallest elements of this set ${\tilde{d}}_{1}$ and ${\tilde{d}}_{2}$ with respect to the frequency ω inside the band gap and the width *l* of the barriers. One can observe that the optimal cavity width is almost independent from the width of the barriers *l* while it strongly depends on the frequency. This dependence is slightly attenuated when considering thin barriers. This is important for the real design of an harvester, with a fixed cavity width, which should work in a wide range of frequencies.

**Figure 9:**
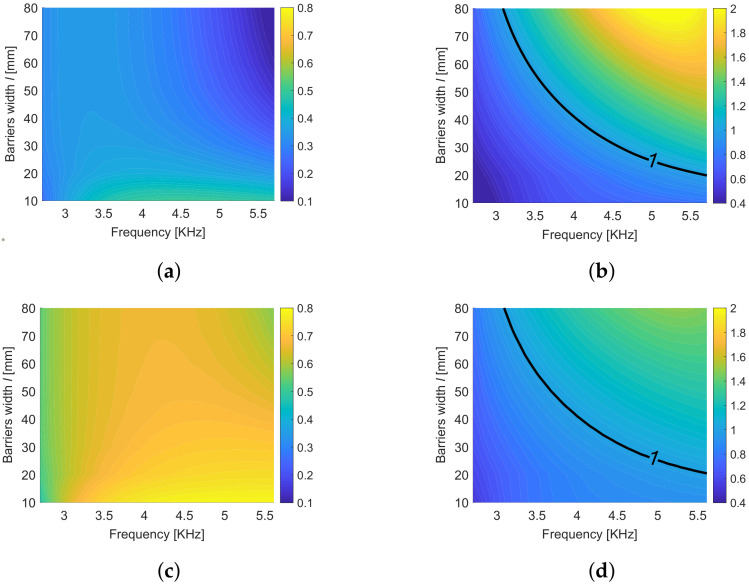
The contours (**a**,**b**) show respectively the indexes IC and the AIC for the first optimal cavity widths $2{\tilde{d}}_{1}$ while (**c**,**d**) for the second optimal cavity widths $2{\tilde{d}}_{2}$. The plots are obtained using analytic expressions.

Keeping the frequency inside the band gap and the barrier width as variables, the two indexes IC and AIC are plotted in Figure 9 using the first two optimal cavity widths $2\;{\tilde{d}}_{1}$ and $2\;{\tilde{d}}_{2}$. The index IC (panels Figure 9a,c) which measures the quantity of energy localized in the cavity with respect to the global energy in the metamaterial with the cavity is higher when considering the larger cavity ($2\;{\tilde{d}}_{2}$). The dependence on the width of the barriers *l* is very limited when considering a frequency close to the band gap opening, while IC decreases with *l* when considering a higher frequency, close to the band gap closing. A different pattern is exhibited by the index AIC (Figure 9b,d) which measures the density of energy localized in the cavity with respect to mean energy density in the metamaterial with the cavity. Values of AIC greater than one correspond to systems effectively concentrating energy in the cavity. High concentration is obtained, for both cavity dimensions, considering high frequency and large barriers; the smaller cavity gives a higher concentration (see Figure 9b,d).

As stated before, a change of the geometry of the unit cell, in terms of both filling fraction and coating thickness, would change the expression of the effective mass density (hence the frequencies of the band gap). Moreover, a change in the filling fraction (or equivalently in the ratio between the radius $R_{c}$ and the cell size *L*) would also modify the value of the effective stiffness of the barriers. To explore the effect of these variations on the energy localization of the system, we study, in particular, the four cases specified in Table 2.

**Figure 10:**
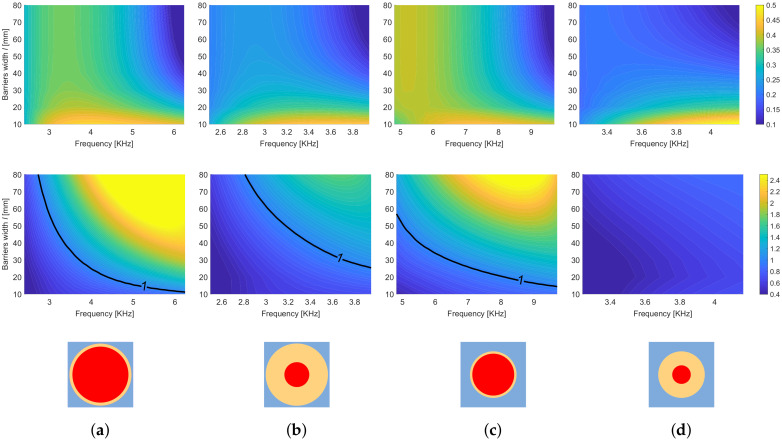
Each panel (**a**–**d**) shows the index IC on the first row and AIC on the second row, for the first optimal cavitywidths $2{\tilde{d}}_{1}$. The following geometrical dimensions are used: (**a**) $R_{c}=0.475L,R_{f}=0.9R_{c},$ (**b**) $R_{c}=0.475L,R_{f}=0.4R_{c},$ (**c**) $R_{c}=0.355L,R_{f}=0.9R_{c},$ and (**d**) $R_{c}=0.355L,R_{f}=0.4R_{c}$.

Figure 10 shows the effect of the geometric modifications of the cell on the indexes IC and AIC; the cavity width is fixed to $2{\tilde{d}}_{1}$ in all cases. Note that the change of the cell also affects the band gap frequencies. In Figure 10 the represented frequency range always corresponds to the first band gap of the LRM. By comparing Figure 10a,b with Figure 10c,d, one can observe that the concentration of energy is improved by increasing the filling fraction of the LRM. At equal filling fraction, a smaller thickness of the coating (Figure 10a,c) also leads to a higher concentration with respect to the case with a large thickness of the coating (Figure 10b,d, respectively).

## 5. Conclusions

This paper investigates the possibility to localize the vibration mechanical energy in a cavity between two barriers constituted by ternary LRMs. The concentration of energy is possible for waves having frequency inside the bandgap of the LRM. The proposed system can be reduced to a one-dimensional problem of wave propagation, thus allowing for a complete analytic solution. We consider the long-wave length regime, and we apply the asymptotic homogenization to obtain the dynamic effective properties of the LRM. The optimal dimension of the cavity for energy localization is given in close form. Results are applied to a specific LRM and validated by comparison with numerical results. The analytic solution allows to highlight the influence of the different material and geometric parameters of the metamaterial on the energy concentration. In particular it is shown that, high filling fraction and small coating thickness of the LRM, besides resulting in a larger band gap at low frequency as shown in [24], improve the energy concentration.

The results obtained in this paper could represent a first step to design a REH with optimal performance in terms of localization of the mechanical energy carried by waves within a specific frequency interval.

The analysis was here restricted to the out-of-plane wave propagation, the case of in-plane propagation, even though leading to more complex expressions, could be treated in a similar way.

The treatment of a real REH, with finite out-of-plane dimension, deserves further studies, as the decoupling between the two cases does not hold anymore. The experimental validation should then be carried out.

## Figures and Tables

**Table 1:** Material properties.

Material	E [MPa]	ν	ρ [Kg/m^3^]
Epoxy	3600	0.370	1180
Rubber	0.118	0.469	1300
Lead	14000	0.420	11340

**Table 2:** Chosen geometry for the unit cell.

Case	$R_{c}/L$	$R_{f}/R_{c}$
a	0.475	0.9
b	0.475	0.4
c	0.355	0.9
d	0.355	0.4

## Appendix A. Dynamic Effective Properties of the LRM

In the sub-wavelength regime, the motion problem of the ternary LRM is characterized by a macroscopic scale x and by a microscopic scale y=x/ϵ, see [21,24,27]. This means that both the displacement field u and the stress field σ can be expanded in the following way:

(A1) $$\begin{array}{cc} u(x) & =u^{0}\left(x,x/\epsilon \right)+\epsilon u^{1}\left(x,x/\epsilon \right)+\epsilon^{2}u^{2}\left(x,x/\epsilon \right)+\cdots \; \end{array}$$

(A2) $$\begin{array}{cc} \sigma (x) & =\sigma^{0}\left(x,x/\epsilon \right)+\epsilon \sigma^{1}\left(x,x/\epsilon \right)+\epsilon^{2}\sigma^{2}\left(x,x/\epsilon \right)+\cdots \end{array}$$

By substituting Equations (A1) and (A2) and their derivatives in Equation (1), an effective description of the LRM is achieved. The terms of the same order in ϵ are then considered. At order −1 one obtains that the displacement field $u_{m}^{0}\left(x,x/\epsilon \right)$ of the matrix is independent from the microscopic variable and will be denoted as $U^{0}\left(x\right)$. At the leading order, and considering the symmetry of the lattice, the homogenized problem is thus governed by the following equation:

(A3) $$\mu_{eff}\;\mathrm{div}\;\mathrm{grad}U^{0}\left(x\right)+\rho_{eff}\left(\omega \right)\;\omega^{2}\;U^{0}\left(x\right)=0$$

where $\mu_{eff}$ is the effective shear modulus. This latter is defined as

(A4) $$\mu_{eff}=\frac{1}{\left|Y\right|}\int_{Y_{m}}\mu_{m}\left({\mathrm{grad}}_{y}\chi +e_{1}\right)\cdot \left({\mathrm{grad}}_{y}\chi +e_{1}\right)\;dy$$

where $e_{1}$ is the unit vector of axis $x_{1}$ and χ is the solution of the cell problem, associated to the matrix only

(A5) $$\left\{\begin{array}{c} \mathrm{div}\;{\mathrm{grad}}_{y}\chi =0\;\;\mathrm{in}\;Y_{m}\\ \mu_{m}\left({\mathrm{grad}}_{y}\chi +e_{1}\right)\cdot n=0\;\;\mathrm{on}\;\partial Y_{c}\\ \chi \;\mathrm{periodic},\;\mu_{m}\;{\mathrm{grad}}_{y}\chi \cdot n\;\text{anti-periodic}\;\mathrm{on}\;\partial Y \end{array}\right.$$

Note that for a general case of a 2D lattice, without the symmetries of the present case, an effective 2×2 stiffness matrix intervenes in Equation (A3) whose entries are defined similarly to Equation (A4) by introducing the solution $\chi^{2}$ of a second cell problem (with $e_{2}$ instead of $e_{1}$ in Equation (A5)).

In Equation (A3), $\rho_{eff}\left(\omega \right)$ is the effective mass density, given by:

(A6) $$\rho_{eff}\left(\omega \right)=\rho_{m}\frac{|Y_{m}|}{\left|Y\right|}+\rho_{f}\frac{|Y_{f}|}{\left|Y\right|}\eta \left(R_{f}\right)+\frac{\rho_{c}}{\left|Y\right|}2\pi \int_{R_{f}}^{R_{c}}\eta \left(r\right)dr$$

where the function η(r) reads:

(A7) $$\eta \left(r\right)=\frac{k_{c}R_{f}\rho_{f}\left(J_{0}\left(k_{c}r\right)Y_{0}\left(k_{c}R_{f}\right)-Y_{0}\left(k_{c}r\right)J_{0}\left(k_{c}R_{f}\right)\right)\;+2\rho_{c}\left(Y_{0}\left(k_{c}r\right)J_{1}\left(k_{c}R_{f}\right)-J_{0}\left(k_{c}r\right)Y_{1}\left(k_{c}R_{f}\right)\right)}{k_{c}R_{f}\rho_{f}\left(J_{0}\left(k_{c}R_{c}\right)Y_{0}\left(k_{c}R_{f}\right)-Y_{0}\left(k_{c}R_{c}\right)J_{0}\left(k_{c}R_{f}\right)\right)\;+2\rho_{c}\left(Y_{0}\left(k_{c}R_{c}\right)J_{1}\left(k_{c}R_{f}\right)-J_{0}\left(k_{c}R_{c}\right)Y_{1}\left(k_{c}R_{f}\right)\right)}$$

where $J_{0}$ and $J_{1}$ are Bessel functions of the first kind, $Y_{0}$ and $Y_{1}$ are Bessel functions of the second kind and $k_{c}=\omega \sqrt{\rho_{c}/\mu_{c}}$.

Denoting by $\omega_{n}$ the set of frequencies which make the denominator of Equation (A7) equal to zero, the Equation (A6) tends to −∞ and to +∞ when ω tends to $\omega_{n}$ from above and from below respectively. The intervals where the effective mass is negative represent band gaps, i.e., intervals of frequencies where waves traveling across the metamaterial are attenuated.
